# Synaptic Plasticity Depends on the Fine-Scale Input Pattern in Thin Dendrites of CA1 Pyramidal Neurons

**DOI:** 10.1523/JNEUROSCI.2071-19.2020

**Published:** 2020-03-25

**Authors:** Ádám Magó, Jens P. Weber, Balázs B. Ujfalussy, Judit K. Makara

**Affiliations:** ^1^Laboratory of Neuronal Signaling, Institute of Experimental Medicine, Hungarian Academy of Sciences, 1083 Budapest, Hungary, and; ^2^János Szentágothai School of Neurosciences, Semmelweis University, 1085 Budapest, Hungary

**Keywords:** dendritic integration, hippocampus, LTP, spine, synaptic plasticity, two-photon uncaging

## Abstract

Coordinated long-term plasticity of nearby excitatory synaptic inputs has been proposed to shape experience-related neuronal information processing. To elucidate the induction rules leading to spatially structured forms of synaptic potentiation in dendrites, we explored plasticity of glutamate uncaging-evoked excitatory input patterns with various spatial distributions in perisomatic dendrites of CA1 pyramidal neurons in slices from adult male rats. We show that (1) the cooperativity rules governing the induction of synaptic LTP depend on dendritic location; (2) LTP of input patterns that are subthreshold or suprathreshold to evoke local dendritic spikes (d-spikes) requires different spatial organization; and (3) input patterns evoking d-spikes can strengthen nearby, nonsynchronous synapses by local heterosynaptic plasticity crosstalk mediated by NMDAR-dependent MEK/ERK signaling. These results suggest that multiple mechanisms can trigger spatially organized synaptic plasticity on various spatial and temporal scales, enriching the ability of neurons to use synaptic clustering for information processing.

**SIGNIFICANCE STATEMENT** A fundamental question in neuroscience is how neuronal feature selectivity is established via the combination of dendritic processing of synaptic input patterns with long-term synaptic plasticity. As these processes have been mostly studied separately, the relationship between the rules of integration and rules of plasticity remained elusive. Here we explore how the fine-grained spatial pattern and the form of voltage integration determine plasticity of different excitatory synaptic input patterns in perisomatic dendrites of CA1 pyramidal cells. We demonstrate that the plasticity rules depend highly on three factors: (1) the location of the input within the dendritic branch (proximal vs distal), (2) the strength of the input pattern (subthreshold or suprathreshold for dendritic spikes), and (3) the stimulation of neighboring synapses.

## Abstract (precis)

Coordinated long-term plasticity of nearby excitatory synaptic inputs has been proposed to shape experience-related neuronal information processing. To elucidate the induction rules leading to spatially structured forms of synaptic potentiation in dendrites, we explored plasticity of glutamate uncaging-evoked excitatory input patterns with various spatial distributions in perisomatic dendrites of CA1 pyramidal neurons in slices from adult male rats.

## Introduction

Cortical pyramidal cells (PCs) receive complex afferent synaptic input patterns that shape somatic action potential (AP) firing. Electrical and biochemical interactions between these correlated inputs are constrained by the complex branching structure of the dendritic tree. Active dendrites allow nonlinear amplification of spatiotemporally correlated inputs by generating various types of d-spikes (Polsky et al., 2004; Losonczy and Magee, 2006; Losonczy et al., 2008; Larkum et al., 2009). Based on *in vitro* experiments, individual dendrites are often considered as distinct integration compartments because branchpoints limit the propagation of regenerative voltage signals, such as dendritic Na^+^ spikes, into other branches (Losonczy and Magee, 2006; Losonczy et al., 2008). Shorter dendritic segments, with a group of coactive inputs, may also represent integration compartments by producing local NMDAR-mediated spikes (Polsky et al., 2004). Furthermore, imaging studies revealed spatially restricted Ca^2+^ signals *in vivo*, suggesting locally correlated synaptic activity (Kleindienst et al., 2011; Takahashi et al., 2012; Cichon and Gan, 2015; Iacaruso et al., 2017; Scholl et al., 2017; Sheffield et al., 2017; Kerlin et al., 2019).

Dendritic compartments may set the rules not only for input–output transformation but also for long-term synaptic plasticity. Although Hebbian long-term plasticity classically considers APs as the global backpropagating signal for LTP and LTD, recent results suggest that the local dendritic response is an important determinant of synaptic plasticity. Supporting this idea, *in vivo* studies revealed that long-term synaptic plasticity related to learning or sensory experience can occur in compartmentalized fashion in dendrites; yet, whether these compartments correspond to integration compartments is unresolved. Some studies showed that newly potentiated or formed synapses are enriched in specific dendrites (Yang et al., 2014; Zhang et al., 2015), pointing to the entire branch as a plasticity compartment. Other reports demonstrated correlated LTP of small clusters of synapses (Makino and Malinow, 2011; Fu et al., 2012; Zhang et al., 2015; Frank et al., 2018), particularly distally along dendrites (Makino and Malinow, 2011), suggesting that the plasticity compartment can be substantially smaller than a dendritic branch. These results indicate that synaptic plasticity depends on various factors available locally at the synapse (e.g., activity of the synapse, local dendritic membrane potential, or Ca^2+^ concentration); thus, synaptic plasticity rules can only be comprehensively understood if the impact of these local variables is elucidated.

The link between the interactions of multiple synaptic inputs and their correlated long-term plasticity locally in the dendrite is however still elusive (Cichon and Gan, 2015; Winnubst et al., 2015; Weber et al., 2016), with several cooperative mechanisms potentially being involved. It is plausible that strong local depolarization by voltage integration of coincident inputs can induce LTP. This may be achieved by d-spikes (Golding et al., 2002; Gordon et al., 2006; Remy and Spruston, 2007; Larkum and Nevian, 2008; Kim et al., 2015), but voltage integration in a range subthreshold to local (or somatic) spikes may also be sufficient. Indeed, we reported that repetitive coactivation of four closely located synapses in distal segments of CA1PC dendrites can induce LTP in the absence of spikes; this subthreshold LTP was input-specific, NMDAR-dependent, and likely underlied by cooperative amplification of NMDAR-mediated Ca^2+^ signals (Weber et al., 2016). Other biochemical factors may also promote spatially clustered synaptic plasticity, e.g., molecules produced by potentiated synapses can induce metaplasticity at nearby synapses active in longer time windows (Harvey and Svoboda, 2007; Govindarajan et al., 2011; Redondo and Morris, 2011).

Little is known about how the above mechanisms are activated by different fine-scale input patterns and how they are affected by local dendritic properties and voltage integration, mainly due to the limitations of most techniques probing synaptic function to control the number and location of the activated synapses. Filling this gap, we combined multisite two-photon glutamate uncaging (2PGU) with two-photon imaging (2PI) and somatic patch-clamp recordings in acute slices to investigate the location- and input-pattern dependence of synaptic plasticity in the perisomatic dendritic arbor of CA1PCs.

## Materials and Methods

### 

#### 

##### Hippocampal slice preparation and patch-clamp recordings.

Adult male Wistar rats (7–11 weeks old) were used to prepare transverse slices (400 μm) from the hippocampus similarly to that described previously (Weber et al., 2016), according to methods approved by the Animal Care and Use Committee of the Institute of Experimental Medicine of the Hungarian Academy of Sciences, and in accordance with the Institutional Ethical Codex, Hungarian Act of Animal Care and Experimentation (1998, XXVIII, section 243/1998) and European Union guidelines (86/609/EEC/2 and 2010/63/EU Directives). Animals were deeply anesthetized with 5% isoflurane and quickly perfused through the heart with ice-cold cutting solution containing the following (in mm): sucrose 220, NaHCO_3_ 28, KCl 2.5, NaH_2_PO_4_ 1.25, CaCl_2_ 0.5, MgCl_2_ 7, glucose 7, Na-pyruvate 3, and ascorbic acid 1, saturated with 95% O_2_ and 5% CO_2_. The brain was quickly removed, and slices were prepared in cutting solution using a vibratome (Vibratome, or VT1000A, Leica Biosystems). Slices were incubated in a submerged holding chamber in ACSF at 35°C for 30 min and then stored in the same chamber at room temperature. For recording, slices were transferred to a custom-made submerged recording chamber under the microscope where experiments were performed at 32°C-35°C in ACSF containing the following (in mm): NaCl 125, KCl 3, NaHCO_3_ 25, NaH_2_PO_4_ 1.25, CaCl_2_ 1.3, MgCl_2_ 1, glucose 25, Na-pyruvate 3, and ascorbic acid 1, saturated with 95% O_2_ and 5% CO_2_.

Cells were visualized using an Olympus BX-61 or a Carl Zeiss Axio Examiner epifluorescent microscope equipped with differential interference contrast optics under infrared illumination and a water-immersion lens (60×, Olympus; or 63×, Carl Zeiss). Current-clamp whole-cell recordings from the somata of hippocampal CA1PCs were performed using a BVC-700 (Dagan) or an EPC800 (HEKA) amplifier in the active “bridge” mode, filtered at 3–5 kHz, and digitized at 50 kHz. Patch pipettes (2–6 mΩ) were filled with a solution containing the following (in mm): K-gluconate 134, KCl 6, HEPES 10, NaCl 4, Mg_2_ATP 4, Tris_2_GTP 0.3, phosphocreatine 14, pH 7.25, complemented with AlexaFluor-488 (100 μm). Series resistance was <30 mΩ. Voltages were not corrected for liquid junction potential. Only CA1PCs with a resting membrane potential (V_rest_) more negative than −55 mV were used. Cells were kept at −63 to −65 mV.

##### Two-photon imaging and uncaging.

A dual-galvanometer-based two-photon scanning system (Prairie Technologies) was used to image the neurons and to uncage glutamate at individual dendritic spines. Two ultrafast pulsed laser beams (Chameleon Ultra II, Coherent) were used: one for imaging of fluorophore (at 920 or 860 nm) and the other to photolyse MNI-caged l-glutamate at 720 nm (Tocris Bioscience; 10 mm), applied through a puffer pipette with an ∼20–30 μm diameter, downward-tilted aperture above the slice, using a pneumatic ejection system (PDES-02TX (NPI). Laser beam intensity was independently controlled with electro-optical modulators (model 350–80, Conoptics). Emitted light was collected by multi-alkali or GaAsP photomultipliers (Hamamatsu Photonics).

All neurons included in the study had mostly complete apical and basal dendritic arbors, with no major dendrites cut. The selected basal (stratum oriens; 65% of all experiments) and apical oblique (proximal stratum radiatum; 35% of all experiments) dendrites were carefully examined and only intact branches with >70 μm length were used. Individual spines with an average phenotype and separated from their neighbors were selected for stimulation. Stimulation was performed by uncaging glutamate ≤0.5 μm lateral to the head of visually identified spines, using 0.5 ms uncaging duration. The uncaging points were placed more than ∼1.1 μm apart (spines on opposing sides of the dendrite could be stimulated individually). Time interval between the stimulated spines in a trace (termed interspine stimulus interval) was 200 ms (for recording individual voltage responses of spines) or 0.1 ms (quasi-synchronous activation during the LTP protocol). Unitary EPSPs were measured repeatedly (usually 6–12 times, repeated every 5 min).

##### LTP experiments.

To measure changes in synaptic function induced by LTP protocols, we recorded EPSPs evoked by 2PGU in whole-cell current-clamp mode. This allowed us to ensure that the applied uncaging stimuli produced EPSPs in the physiological amplitude range regardless of the depth of the spines, requiring fine adjustments in uncaging laser power in each experiment. Furthermore, in our experience, electrophysiological recordings provide the best way to detect even subtle signs of photodamage to confidently distinguish plasticity-related effects from phototoxicity. Accordingly, experiments showing electrophysiological signs of photodamage (sudden large irregular depolarization with uneven and slow repolarization during LTP protocol with consecutive loss of reliable single spine responses, often accompanied by morphological changes including spine swelling and contour changes or dendritic swelling) were terminated and excluded from the analysis. We chose not to measure fluorescence-based spine volume for monitoring structural LTP (Matsuzaki et al., 2004) because AlexaFluor-488 fluorescence continuously increased in the repatched cells due to dialysis from the patch pipette.

**Figure 1.**
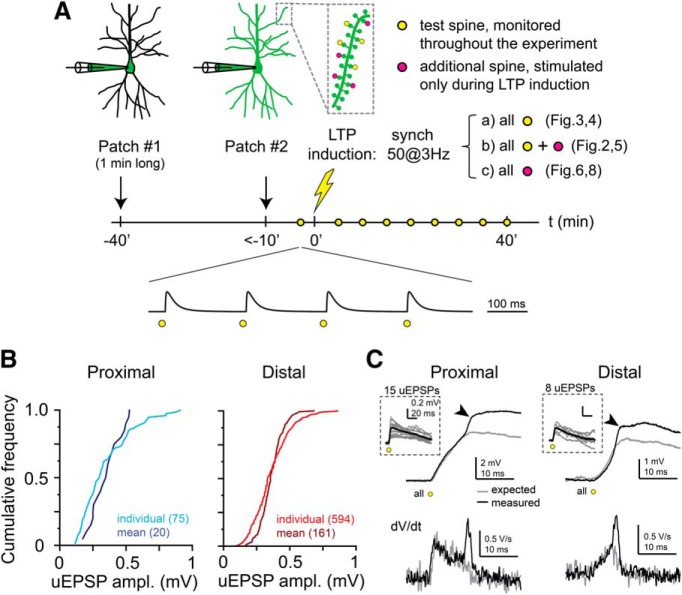
Protocol settings for LTP experiments. ***A***, Schematic of the experimental timeline. ***B***, Cumulative frequency of all individual test spine EPSP amplitudes and the mean EPSP amplitudes of the test spines in individual experiments at proximal (left, blue) and distal (right, red) dendritic locations. ***C***, Representative somatic recordings of d-spikes evoked by quasi-synchronous multisynaptic glutamate uncaging at proximal (left, same trace shown in Fig. 2*B*) and distal (right) segments (in two different cells). Top, Recorded response to the first stimulus during the LTP protocol (black), and the arithmetic sum (gray) of the individual EPSPs (inset). Arrows indicate the fast spikelet mediated by voltage-gated Na^+^ channels. Bottom, dV/dt of the voltage traces. Note the transient dV/dt component associated with the fast Na^+^ spikelet.

To prevent washout of intracellular components by whole-cell dialysis (Matsuzaki et al., 2004), we used a method where LTP protocol could be started within 10 min after establishing the whole-cell configuration, as previously developed (Weber et al., 2016) (see Fig. 1*A*). Neurons (usually 3–4 per slice) were first patched with a pipette solution containing AlexaFluor-488, the cell was dialyzed for 30–60 s (usually facilitated by gently blowing into the pipette), and then the pipette was carefully withdrawn. After 30–100 min, the dye diffused sufficiently to visualize most of the dendritic arborization. A proximal (relative distance along branch: <0.4; total distance from soma: 82 ± 9 μm, *n* = 20) or distal (relative location along branch: >0.6; total distance from soma: 181 ± 4 μm, *n* = 160) fluorescent dendritic segment with well-visible individual spines was selected and a *z* stack was obtained (0.5 μm z-steps). Then, the soma of the same cell was patched again, guided by fluorescent identification using either 2PI or a camera (Andor Zyla). Success rate for repatching exceeded 90%, and repatched neurons had normal V_rest_ (more negative than −55 mV). After establishing whole-cell configuration again and measurement of V_rest_, uncaging started immediately. A set of four individual spines were first stimulated separately (200 ms between spines; trials repeated typically with 0.02–0.5 Hz), and the uncaging laser power was adjusted to yield physiological-sized EPSPs at each stimulated spine (see Fig. 1*B*). Spines that did not respond reliably to uncaging were replaced by new ones until four test spines with relatively uniform EPSP amplitudes were found (as a result of this selection procedure, the final four spines may have received variable numbers of pre-LTP test stimuli). After the test recording, an LTP induction protocol (50 stimulations at 3 Hz at a group of spines, unless otherwise indicated; see Fig. 5*E*,*F*, in various configurations as indicated in the text and figures) was applied as soon as possible (within 2 min from the last test stimulus and within 10 min after break-in). In all homosynaptic LTP experiments, the same laser power was used for the LTP induction protocol as that for monitoring the test spines throughout the experiment. In some of the heterosynaptic LTP experiments (see Figs. 6, 8), the laser power was increased by ∼15% during the LTP induction protocol, to increase the likelihood to evoke d-spikes by the LTP induction spines; then we commenced monitoring the test spines with the original test laser power. The uncaging locations were manually readjusted, if necessary, between test pulses (every 5 min) due to occasional changes in shape, position, or loading-related fluorescence of the stimulated spines. Care was taken not to move the uncaging location closer to the spine head during the experiment, to avoid the possibility of artificial increases in EPSP amplitudes. In experiments where d-spikes were evoked during LTP protocol, we accepted experiments if we could either detect a clear transient rise in the dV/dt (related to dendritic Na^+^ spikes) (Losonczy and Magee, 2006; Losonczy et al., 2008; Makara et al., 2009) or measured at least 2 mV peak nonlinearity comparing the measured compound EPSP to the arithmetic sum of the individual EPSPs (Losonczy and Magee, 2006; Losonczy et al., 2008; Makara et al., 2009). The presence of d-spike(s) by at least one stimulus was always confirmed by visual inspection of the LTP trace by at least two investigators, and in most cases was also evaluated using a semiautomated spike detection algorithm. D-spikes were usually most clearly detected at the first stimulus of the LTP induction protocol. Because in some cases the presence of a fast d-spike was ambiguous at later stimuli (most likely due to partial inactivation of voltage-gated Na^+^ channels) (Remy et al., 2009), we did not attempt to systematically calculate the proportion of stimuli with and without d-spikes in the full dataset.

##### Chemicals.

d-AP5 (Tocris Bioscience) and NiCl_2_ (Sigma Millipore) were dissolved in distilled water; nimodipine and U0126 were dissolved in DMSO. Aliquots of the stock solutions were stored at −20°C and dissolved into ACSF on the day of the experiment. Inhibitors were applied by perfusing the slice with ACSF containing the blocker(s) for 10–15 min before repatching the cells. Solution containing nimodipine and Ni^2+^ was protected from light.

##### Data analysis.

Analysis was performed using custom-written macros in IgorPro (WaveMetrics). Voltage signals were analyzed offline using averaged traces of typically 3–12 trials with no smoothing. Individual traces, where the rising phase or the peak of an uncaging-evoked EPSP was contaminated by spontaneous EPSPs, were not included in the average. Calculated EPSP amplitudes were measured offline as the peak of the arithmetic sum of the individual responses (mimicking the same input timing as used experimentally). Test spines were typically monitored every 5 min. For assessing temporal changes, data were pooled between 5–10, 15–25, and 30–40 min.

The magnitude of plasticity was quantified as the mean normalized change in EPSP amplitude of all test spines, averaged between 30 and 40 min after the LTP protocol. An individual spine was considered to be potentiated with normalized EPSP >1.3 after LTP (Matsuzaki et al., 2004; Weber et al., 2016), which corresponded to a cutoff at 95% of the spines measured in control experiments with no LTP protocol (see Figs. 3*B*,*E*, 6*B*). We did not analyze spines with <0.1 mV initial EPSP amplitude to avoid overestimation of LTP due to division by small numbers. Occasionally (<5%), we observed a retraction or disappearance of the stimulated spine, usually accompanied by a strong reduction (<40% of the control value) or unreliability of response amplitudes. This seemed to occur independently of the location of the spines or the experimental protocol; therefore, we omitted such spines from the analysis. Spines were excluded also if their head moved close to other neighboring spines due to the shape or size changes throughout the course of the experiment. Spines were included in the analysis only if: (1) initial EPSP amplitude was between 0.1 and 1 mV, (2) either all three normalized EPSP amplitudes (at 30, 35, and 40 min) after LTP induction were >1.8 or <0.6, or the SD was <0.35 in case of average change >1 or SD was <0.6 in case of average change ≤1. Experiments with 4 test spines where more than one spine failed to fulfill these criteria were discarded; in experiments using 2 or 3 test spines, all spines fulfilled the criteria.

Morphological and distance measurements were performed on dye-loaded neurons using ImageJ (National Institutes of Health). Distance of input site from the soma or trunk was measured from the approximate midpoint of the input site on stacked images. Interspine distances (ISDs) were measured between spine insertion points to the shaft (either visible or the perpendicular projection of the spine head center to the shaft) on stacks or single-focal images. Relative distances along branch were measured as the distance of the input site center divided by the total branch length, measured from the soma (basal dendrites) or the originating branch point from the trunk (apical oblique dendrites). In cases when the dendrite bifurcated distal to the input site (e.g., proximal stimulation sites), the longer daughter was measured for total branch length.

##### Computational modeling.

We used a detailed biophysical CA1 PC model (Ujfalussy and Makara, 2020) based on Jarsky et al. (2005), optimized for reproducing the dendritic processing of synaptic inputs in CA1 pyramidal neurons (Losonczy and Magee, 2006).

The default passive parameters of the model were as follows: C_m_ = 1 μF/cm^2^, R_a_ = 100 Ωcm and R_m_ = 20 kΩcm^2^ in the dendrites, R_m_ = 40 kΩcm^2^ in the soma and in the axon, and R_m_ = 50 kΩcm^2^ in the axonal nodes. Activated synapses were placed on high-impedance dendritic spines consisting of a spine neck (length: 1.58 μm; diameter: 0.077 μm) and spine head (length: 0.5 μm; diameter: 0.5 μm) with total neck resistance ∼500 mΩ (Harnett et al., 2012). To correct for the presence of spines, C_m_ was increased and R_m_ was decreased by a factor of 2 in dendritic compartments beyond 100 μm from the soma. In the simulations shown in Figure 7*F*, we increased R_a_ to 200 Ωcm (high R_a_) and changed R_m_ to 10 (low R_m_), 20 (medium R_m_), or 40 (high R_m_) kΩcm^2^ in compartments beyond 100 μm from the soma. These manipulations altered Na^+^ spike dV/dt amplitude in a range of 75%–120% and EPSP amplitude in a range of 87%–116% (both parameters still remaining in the physiological range).

Ion channel parameters were adjusted to replicate the most important features of dendritic integration of excitatory synaptic inputs. The model contained voltage-gated Na^+^, K_DR_, and K_A_ channels with the following densities (all in S/cm^2^): Na^+^: axon initial segment: 15; soma: 0.2; dendrites: 0.03 and increasing from 0.04 to 0.06 S/cm^2^ along the apical trunk between 100 and 500 μm. K_DR_: axon, soma, and apical trunk: 0.04; all other dendritic branches: 0.02. K_A_: axon: 0.004; soma and dendritic branches: 0.02; and increasing from 0.048 to 0.29 along the apical trunk between 100 and 500 μm.

The model included AMPA and NMDA excitation with synaptic conductances modeled as double-exponential functions with the following parameters: AMPA: τ_1_ = 0.1 ms, τ_2_ = 1 ms, g_max_ = 0.6 nS and E_rev_ = 0 mV; NMDA: τ_1_ = 2 ms, τ_2_ = 50 ms, g_max_ = 0.8 nS, and E_rev_ = 0 mV. The voltage dependence of the NMDA conductance was captured by a sigmoidal activation curve: g_NMDA_ = g_0_ (1 + C_Mg_/4.3 exp(−0.071 V))^−1^ where V is the local dendritic membrane potential and C_Mg_ = 1 mm is the Mg^2+^ concentration.

The model captures several somatic and dendritic properties of these cells measured under *in vitro* conditions, including the generation and propagation of Na^+^ APs at the soma and along the apical dendritic trunk; the generation of local Na^+^ spikes in thin dendritic branches; amplitude distribution of synaptic responses; nonlinear integration of inputs via NMDARs; the similar voltage threshold for Na^+^ and NMDA nonlinearities; and the major role of A-type K^+^ channels in limiting dendritic excitability. When stimulated with *in vivo*-like synaptic inputs distributed throughout the entire dendritic tree, the same biophysical model shows place-selective activity, with several features of the somatic membrane potential activity falling in the physiological range (Ujfalussy and Makara, 2020). The simulations were performed with the NEURON simulation environment (version 7.4) embedded in Python 2.7.

##### Experimental design and statistical analysis.

No statistical methods were used to predetermine sample sizes, but our samples are similar to or exceed those reported in previous publications and that generally used in the field. Experiments were not blind. Statistical analysis was performed using Wilcoxon matched pairs test (two paired groups), one-sample Wilcoxon test (some analysis of LTP experiments, comparison to median = 1), Mann–Whitney *U* test (two unpaired groups), Kruskal–Wallis test and *post hoc* multiple comparisons with Holm-Bonferroni adjustment (multiple unpaired groups), or χ^2^ test (comparing proportions) using Statistica (Statsoft) software. The specific test used for a given analysis is indicated in the text. All statistical tests were two-tailed. Differences were considered significant when *p* < 0.05. Data are reported as mean ± SEM, unless otherwise indicated.

Each LTP experiment was performed in a different cell in a different slice, typically in different animals on different days. One or two experiments per animal were performed. *N* represents the number of experiments or number of spines as indicated.

##### Data availability.

The data that support the findings of this study are available from the corresponding author upon reasonable request.

## Results

### Regenerative d-spikes are required for efficient cooperative LTP at proximal dendritic locations

To investigate plasticity of various synaptic input patterns, we used short 2PGU pulses at multiple individual spines with laser powers producing physiological-like individual EPSPs (Fig. 1*B*). We selected a set of four “test” spines at either proximal (relative location <0.4) or distal locations (>0.6) along individual branches, whose EPSPs were monitored by asynchronous stimulation (interspine time interval, 200 ms) throughout the experiment (Weber et al., 2016) (Fig. 1*A*; see Materials and Methods). After measuring physiologically adjusted baseline EPSP amplitudes (Fig. 1*B*), we applied an LTP induction protocol consisting of 50 quasi-synchronous (interspine stimulus interval, 0.1 ms) stimulation of selected spine groups (Fig. 1*A*; input configurations explained below) at 3 Hz. The mode of voltage integration during the LTP induction protocol, specifically the presence of d-spikes, was evaluated by comparing the amplitude and kinetics of the expected and measured compound EPSPs (Fig. 1*C*; see Materials and Methods). After the LTP protocol, EPSP amplitudes of the test spines were monitored for 30–40 min.

**Figure 2.**
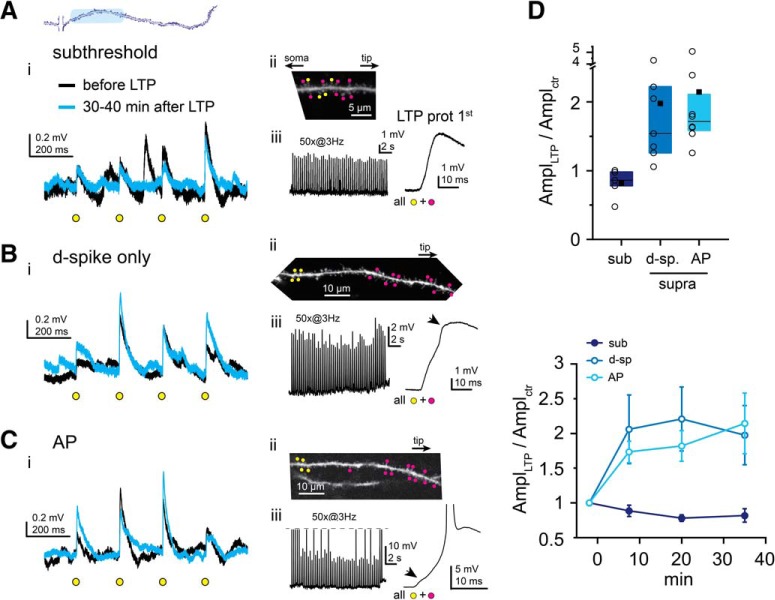
d-spikes are required for cooperative LTP at proximal dendritic locations. ***A***, Top, Schematic of an individual dendrite. Blue represents proximal area. ***A–C***, Representative recordings using clustered subthreshold (***A***), dendritically suprathreshold (***B***), and somatically suprathreshold (***C***) input patterns. ***Ai***, ***Bi***, ***Ci***, Representative recordings (average traces) of individual EPSPs evoked by 2PGU (yellow circles) at the four test spines before (black) and >30 min after (blue) the LTP protocol. ***Aii***, ***Bii***, ***Cii***, 2P images (*z* stack or single plane) of the stimulated segments. Yellow dots indicate test spine 2PGU sites. Magenta dots indicate additional spines costimulated with test spines during the LTP protocol. ***Aiii***, ***Biii***, ***Ciii***, LTP induction protocol trace (left), and the first stimulus enlarged (right). Arrows point to dendritic Na^+^ spikes as detected at the soma. APs are truncated in ***Ciii***. ***D***, Summary of LTP experiments at proximal dendritic segments. Top, Mean (square), median (line), and interquartile ranges (box) of EPSP amplitude changes at 30–40 min relative to baseline. Circles represent data (mean of the four test spines) in individual experiments. Bottom, Time course of EPSP amplitude changes (mean ± SEM of experiments).

We first focused on cooperative LTP induction by synapses located at proximal sites along perisomatic dendrites (Fig. 2). In our previous study, at these locations we observed no subthreshold LTP after coactivation of four clustered test synapses during the LTP protocol (Weber et al., 2016). We therefore first asked whether larger clusters of proximal inputs were able to evoke subthreshold LTP. We increased the synapse cluster costimulated during the LTP protocol to 12–16 inputs by uncaging at additional neighbor spines together with the test spines, covering ∼10–15 μm (Fig. 2*Aii*). Synchronous stimulation of such a sizable synapse cluster evoked substantial somatic EPSPs (first EPSP during LTP protocol: 3.9 ± 0.5 mV, *n* = 5 experiments; Fig. 2*Aiii*) with small peak EPSP nonlinearity (0.7 ± 0.5 mV, measured in *n* = 4 experiments), but regenerative d-spikes were not triggered (Fig. 2*Aiii*; see Materials and Methods), likely due to the low impedance of proximal dendritic segments (Harnett et al., 2012). Surprisingly, even with this high local input density, with the subthreshold pattern no LTP was observed at the test synapses; indeed, the mean EPSP amplitude of test spines tended to rather decrease (Fig. 2*Ai*,*D*; EPSP amplitude relative to baseline at 30–40 min: 0.82 ± 0.09, mean ± SEM of *n* = 5 experiments, *p* = 0.079, one-sample Wilcoxon test).

We next asked whether proximal synapses can undergo LTP if they contribute to input patterns triggering d-spikes. D-spikes in thin perisomatic dendrites comprise fast Na^+^ spikes and/or slow NMDA spikes (Losonczy and Magee, 2006; Losonczy et al., 2008). To facilitate d-spike initiation, we costimulated the four proximal test spines during the LTP protocol with a group of 11 additional spines located more distally on the same dendrite (∼25 μm distance between group centers; Fig. 2*B*). This input pattern evoked d-spikes more efficiently, likely due to extending to higher impedance dendritic segments. Generation of d-spikes was indicated by either a transient increase in the rate of rise (dV/dt) of the compound EPSPs, a sign of dendritic Na^+^ spikes (Losonczy and Magee, 2006; Losonczy et al., 2008) (Figs. 1*C*, 2*Biii*), and/or a peak somatic nonlinearity ≥2 mV, indicating NMDA spikes (Losonczy and Magee, 2006; Losonczy et al., 2008). We prevented somatic APs by slight hyperpolarization during the LTP protocol. Triggering d-spikes induced robust long-lasting increase in the mean EPSP amplitude of the four proximal test spines (1.98 ± 0.43, *n* = 7 experiments; Fig. 2*B*,*D*; different from subthreshold with *p* = 0.024, multiple comparisons after Kruskal–Wallis test with *p* = 0.004), and potentiated synapses were found in every experiment. Similar LTP was measured when APs were also evoked by at least 1 of the 50 LTP stimulus pulses: EPSP amplitude increased to 2.15 ± 0.44 (Fig. 2*C*,*D*; *n* = 8 experiments, different from subthreshold with *p* = 0.004, not different from d-spike only, *p* = 1, multiple comparisons after Kruskal–Wallis test with *p* = 0.004), and potentiated synapses were found in all experiments. These data suggest that large depolarization, involving regenerative dendritic spikes (local or backpropagating AP), is needed for cooperative LTP induction of synapses located in proximal segments of perisomatic dendrites.

### Subthreshold LTP at distal dendritic locations depends on fine-scale input configuration

**Figure 3.**
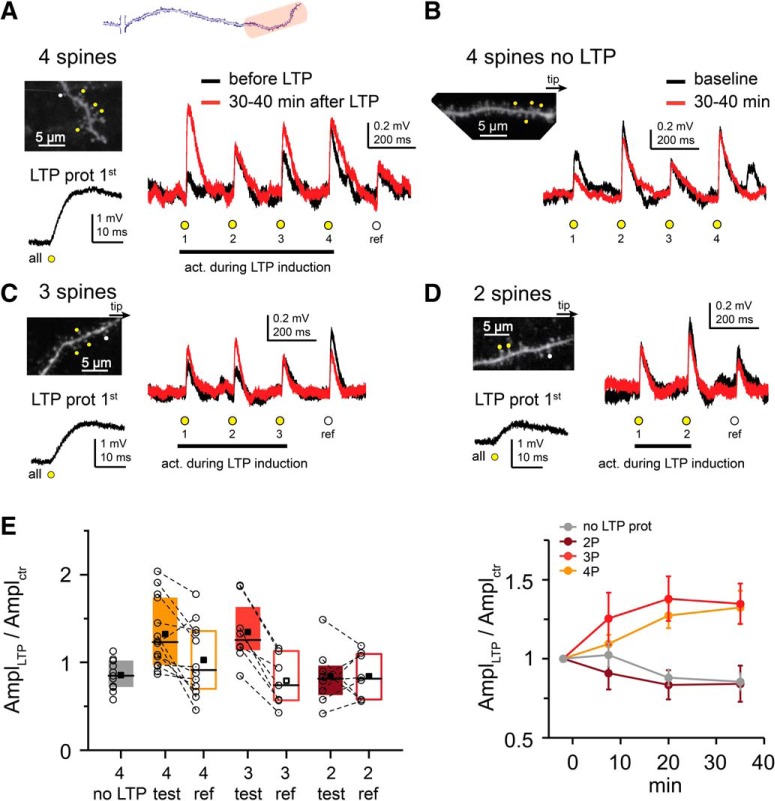
Cluster size requirements for subthreshold cooperative LTP at distal dendritic locations. ***A***, Top, Schematic of an individual dendrite, with stimulated distal area indicated in red. ***A–D***, Representative experiments. ***A***, Left, Top, 2P image of the distal segment of a perisomatic dendrite. Test spines marked by yellow dots were costimulated in the LTP protocol; the reference spine (white dot) was not. Left, Bottom, First stimulus of the LTP protocol. Right, Somatic EPSPs by the five spines (s1–4, yellow circles; and reference spine, white circle), before (black) and >30 min after (red) the LTP protocol. ***B***, Experiment where four test spines (yellow dots in 2P image) were monitored but no LTP protocol was applied. ***C***, ***D***, Similar experiment as in ***A***, but with only 3 (***C***) or 2 (***D***) test spines stimulated during the LTP protocol. ***E***, Summary of experiments with clustered distal spines. Circles represent mean data of the test spines or data from individual reference spines in each experiment. Test and reference spine data from the same experiment are connected. Note that data from individual (reference) spines have inherently higher variance than that of mean data of multiple (test) spines. Right, Time course of EPSP amplitude changes in test spines (mean ± SEM).

We next explored the rules of cooperative LTP at distal segments of perisomatic dendrites. Similarly to our previous report (Weber et al., 2016), repeated coactivation of four clustered distal test spines (dendritic stretch: 4.59 ± 0.25 μm) increased their EPSP amplitude to 1.32 ± 0.11 (*n* = 14 experiments; Fig. 3*A*,*E*), and potentiation occurred in at least one spine in most (11 of 14) experiments (Fig. 3*A*). D-spikes were not detected during the LTP protocol (Fig. 3*A*; for criteria, see Materials and Methods). Subthreshold LTP was specific to the coactive inputs because single “reference” spines (a fifth synapse that was only monitored, but was not stimulated during LTP induction) showed, on average, no change in EPSP amplitude (1.03 ± 0.12 in *n* = 12 experiments; comparison with test spines: *p* = 0.012, Wilcoxon test; Fig. 3*E*). In control experiments where EPSPs of four test spines were monitored without coactivation (no LTP protocol), EPSPs did not increase, indeed slightly decreased (Fig. 3*B*,*E*; EPSP amplitude: 0.86 ± 0.05, *n* = 11, *p* = 0.029, one-sample Wilcoxon test; comparison of experiments with and without LTP protocol: *p* = 0.001, Mann–Whitney test).

To determine the minimum cluster size required for subthreshold cooperative LTP, we reduced the number of test synapses coactivated during LTP induction. With three synapses, we still observed input-specific increase in EPSP amplitude (1.35 ± 0.13, *n* = 8; Fig. 3*C*,*E*), similar to that measured with four inputs. In contrast, LTP protocol with only two synapses did not induce their potentiation (0.84 ± 0.11, *n* = 8; Fig. 3*D*,*E*); their EPSP amplitude changes were similar to those of reference spines (0.84 ± 0.09, *n* = 8, *p* = 0.89, Wilcoxon; Fig. 3*E*). Statistical analysis showed similar LTP with coactivation of 3 or 4 spines (*p* = 1), which was larger than that with 2 spines (*p* = 0.025 and *p* = 0.018, respectively, multiple comparison test after Kruskal–Wallis ANOVA with *p* = 0.010).

**Figure 4.**
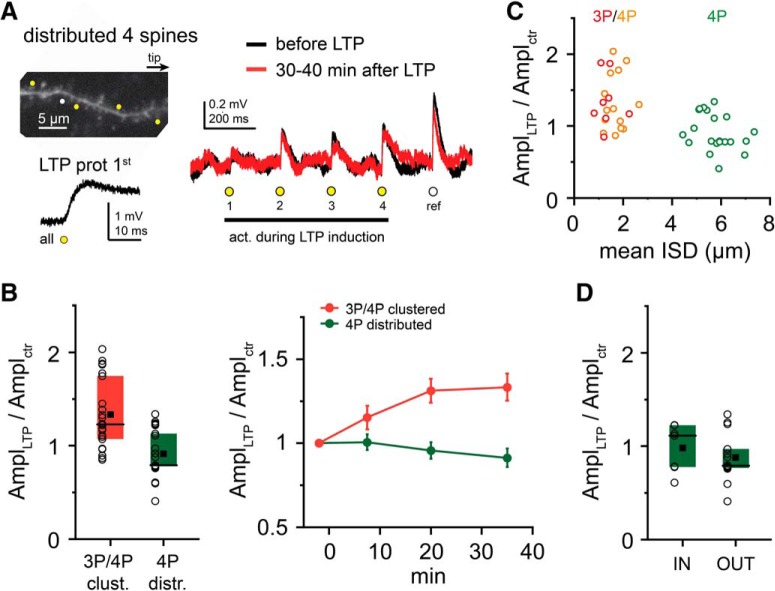
Spatial requirements for subthreshold cooperative LTP at distal dendritic locations. ***A***, Representative experiment similar to Figure 3 but using more distributed test spines. ***B***, Summary of experiments with clustered (experiments with 3 and 4 spines pooled) and distributed arrangements. ***C***, Mean ISD between two neighboring test spines in clustered and distributed arrangements. ***D***, Results of experiments with distributed inputs in tip-to-soma (IN) and soma-to-tip (OUT) sequences.

Next, we examined the spatial pattern requirements for subthreshold LTP. LTP protocol with four coactivated test spines distributed evenly on longer dendritic stretches (17.4 ± 0.5 μm, ISD: 5.8 ± 0.2 μm; Fig. 4*A*,*C*) did not produce subthreshold LTP effectively (EPSP amplitude: 0.91 ± 0.05, *n* = 21, *p* < 0.001 for comparison with tightly clustered arrangement with 3 or 4 spines, Mann–Whitney test; Fig. 4*A–C*; 13% of all spines potentiated, comparison with tightly clustered: *p* < 0.001, χ^2^ test), with strong negative correlation between ISD and EPSP change (Fig. 4*C*; *p* < 0.001, Spearman *r* = −0.548, *n* = 43). No significant difference was found between tip-to-soma (0.98 ± 0.09, *n* = 7) and soma-to-tip (0.88 ± 0.07, *n* = 14) sequences (*p* = 0.681, Mann–Whitney test; Fig. 4*D*). Together, these results show that tight clusters of ≥3 coactive distal inputs can be strengthened by subthreshold cooperative LTP, even without regenerative dendritic activity.

### d-spikes alleviate the tight clustering requirements of LTP

**Figure 5.**
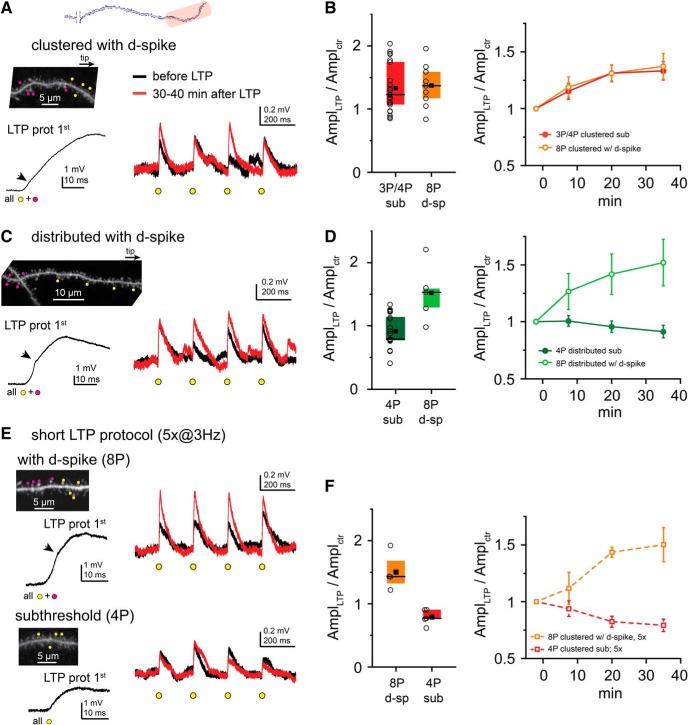
Role of d-spikes in cooperative LTP at distal dendritic locations. ***A***, Representative experiment with clustered input pattern. Left, Top, 2P image of a distal dendritic segment. Four clustered test spines (yellow dots) were costimulated during the LTP protocol with four additional spines (magenta dots) to trigger d-spikes on at least one stimulus. Left, Bottom, First stimulus of the LTP protocol. Right, Somatic EPSPs by the four test spines before (black) and >30 min after (red) the LTP protocol. ***B***, Comparison of LTP with subthreshold (red; pooled data with 3 and 4 spines from Fig. 3*E*) and locally suprathreshold clustered input patterns with 8 spines (orange). Left, Mean (square), median (line), and interquartile ranges (box) of relative EPSP amplitude changes. Circles represent individual experiments (mean data of the four test spines). Right, Time course of EPSP amplitude changes (mean ± SEM of all experiments). ***C***, Similar representative experiment as in ***A***, but with distributed test spine arrangement. ***D***, Comparison of LTP with subthreshold (dark green; data from Fig. 4*B*) and locally suprathreshold (light green) distributed input patterns. ***E***, Representative experiments with short LTP protocol (costimulation 5 times, 3 Hz). Top, Experiment with four clustered test spines and four additional spines coactivated during the short LTP protocol triggering d-spikes. Bottom, Four clustered test spines costimulated in the short LTP protocol, evoking no d-spikes. Right, Somatic EPSPs by the four test spines before (black) and >30 min after (red) the LTP protocol. ***F***, Comparison of the effect of short LTP protocol with subthreshold (red) and locally suprathreshold input patterns (orange). Left, Mean (square), median (line), and interquartile ranges (box) of relative EPSP amplitude changes. Circles represent individual experiments (mean data of the four test spines). Right, Time course of EPSP amplitude changes (mean ± SEM). ***A***, ***C***, ***E***, Arrows indicate dendritic Na^+^ spikes (Figs. 1*C*, 7).

Are the strength and/or the spatial rules of plasticity at distal dendritic segments different if synapses participate in a stronger input pattern that can evoke local d-spikes? To address this question, during the LTP protocol, additional neighbor spines were coactivated together with the four clustered test inputs to trigger d-spikes (Fig. 5*A*). In most cases, eight synchronous synapses were enough to evoke at least one regenerative dendritic event during the LTP protocol without somatic APs (Fig. 5*A*; see also Fig. 1*C*). This clustered, locally suprathreshold input pattern induced LTP in at least one test spine in all experiments (9 of 9). Surprisingly, neither the magnitude of LTP (1.37 ± 0.11, *n* = 9; vs 1.33 ± 0.08, *n* = 22 for subthreshold LTP with 3 or 4 spines, *p* = 0.727, Mann–Whitney test) nor the ratio of potentiated synapses (47% vs 39%, *p* = 0.456, χ^2^ test) was significantly different from that measured with subthreshold LTP by 3 or 4 clustered inputs (Fig. 5*B*).

We next explored how LTP with d-spikes depends on the spatial arrangement of the inputs. We hypothesized that more extended propagation of d-spikes, especially toward the sealed tip, may allow more distributed input patterns to potentiate. To test this, we distributed the four test spines (total stretch: 23.2 ± 2.6 μm, average ISD = 8.3 ± 0.8 μm, *n* = 5), and during LTP induction we coactivated them with four additional (more proximal) synapses to trigger d-spikes (Fig. 5*C*). In most experiments (4 of 5), we found at least one synapse to be potentiated, and an average LTP of 1.52 ± 0.20 was induced (*n* = 5; Fig. 5*C*,*D*). The effect was significantly stronger than that measured with only four distributed (subthreshold) synapses (*p* = 0.004, Mann–Whitney test; Fig. 5*D*).

Previous reports using electrical stimulation indicated that d-spikes can trigger synaptic potentiation with fewer stimulus repetitions than other LTP-inducing activity patterns (Remy and Spruston, 2007; Bittner et al., 2017). To test whether there is a difference in this regard between locally subthreshold and suprathreshold input patterns, we performed experiments with a short LTP protocol, consisting of only 5 coactivations of 4 (subthreshold) or 8 (suprathreshold for d-spikes) clustered spines. We found that suprathreshold clustered inputs did develop robust LTP (1.50 ± 0.15, *n* = 4), whereas only 5 synchronous activations were not sufficient to induce LTP with subthreshold clustered inputs (0.79 ± 0.05, *n* = 5, *p* = 0.019 compared with d-spikes, Mann–Whitney test; Fig. 5*E*,*F*).

These results together indicate that d-spikes, although not necessarily required for LTP at distal dendritic segments, can alleviate the tight spatial clustering requirements and reduce the number of coincident activity events needed to induce cooperative LTP.

### Strong input patterns allow local plasticity crosstalk

**Figure 6.**
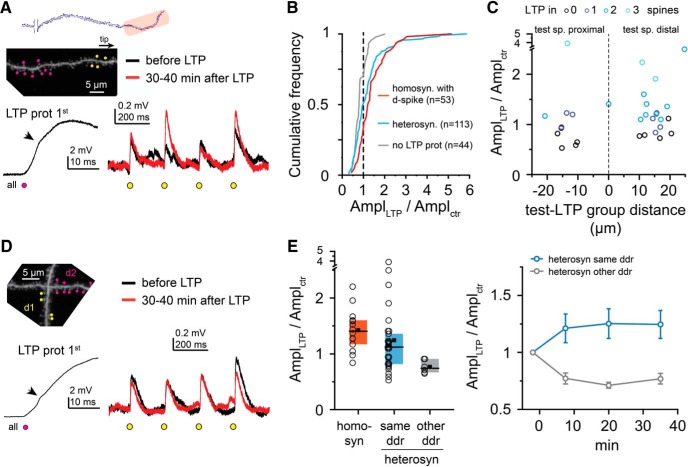
Heterosynaptic plasticity by suprathreshold input patterns at distal dendritic locations. ***A***, Representative experiment. Left, Top, 2P *z* stack image of a distal dendritic segment. Four clustered test spines (yellow dots) were monitored; but during the LTP protocol, only nearby spines (magenta dots) were stimulated to trigger d-spikes on at least one stimulus. Left, Bottom, First stimulus of the LTP protocol. Right, Average somatic EPSPs at the four test spines before (black) and >30 min after (red) the LTP protocol. ***B***, EPSP amplitude changes of individual test spines in homosynaptic (orange; suprathreshold experiments from Fig. 5*B–D*) and heterosynaptic (blue) LTP experiments. Number of spines included in the analysis is indicated in parentheses for each condition. Spines from no LTP experiments (Fig. 3*B*,*E*) are shown in gray for reference. ***C***, Impact of relative dendritic distance between the test spine and LTP induction spine groups. ***D***, Similar as in ***A***, but test spines and LTP induction spines are located on two different dendritic branches of the same cell. ***E***, Left, Comparison of homosynaptic LTP and heterosynaptic LTP, with test spines located on the same dendrite as the LTP induction spines (light blue) or on a nearby other dendrite (gray). Right, Time course of mean EPSP changes in heterosynaptic experiments.

D-spikes, evoking robust voltage and Ca^2+^ signals in the branch (Losonczy and Magee, 2006; Losonczy et al., 2008), may activate signaling mechanisms that affect the function of not only those synapses that evoked them but other neighbor synapses as well. To examine this possibility, we coactivated a group of eight spines during LTP induction, triggering d-spikes (“LTP induction spines”), and measured the impact of this stimulus on EPSP amplitudes of a different set of nearby four test spines (up to ∼20 μm distance; Fig. 6*A*). The test spines were thus only activated before (≤2 min, on average 94 s) and after (≥5 min, on average 369 s), but not during the LTP protocol. Surprisingly, we observed variable effects: although the long-term change in test spine EPSP amplitude (1.25 ± 0.12, *n* = 30) was smaller than that by homosynaptic LTP with d-spikes (clustered and distributed suprathreshold data from Fig. 5*A–D* pooled; 1.43 ± 0.10, *n* = 14, *p* = 0.030, Mann–Whitney test), in a substantial fraction of experiments, we detected signs of potentiation in the test spines (Fig. 6*A*,*B*,*E*). First, in the majority of experiments (20 of 30), EPSP increased >30% in at least one of the test spines (Fig. 6*A*,*B*). Second, in 12 of 30 experiments, the mean EPSP amplitude change in the test spines was larger than the mean +2 SD measured in control experiments with no LTP protocol (Fig. 6*E*; compare with Fig. 3*B*,*E*). This heterosynaptic “crosstalk” potentiation could occur both if the test spines were proximal or distal from the LTP induction spines (Fig. 6*C*).

To better understand the nature of this effect, we tested whether crosstalk potentiation is evoked when test spine and LTP induction spine groups are located at short Eucledian distance, but on different dendrites of the cell (Eucledian distance: 7.1 ± 1.4 μm, dendritic path: 228 ± 19 μm; *n* = 6; Fig. 6*D*). Under these conditions, no LTP was found in any of the test spines, and their EPSP amplitude rather decreased (0.77 ± 0.05, *n* = 6, *p* = 0.013 compared with arrangement with all spines on the same dendrite, Mann–Whitney test; Fig. 6*E*), similar to the control experiments with no LTP protocol (Fig. 3*B*,*E*). This indicates that the crosstalk mechanism involves intracellular rather than extracellular signal(s), and affects only the activated dendrite segment. These results also excluded that the effect could be attributed to glutamate diffusion or other nonspecific effects of 2PGU.

**Figure 7.**
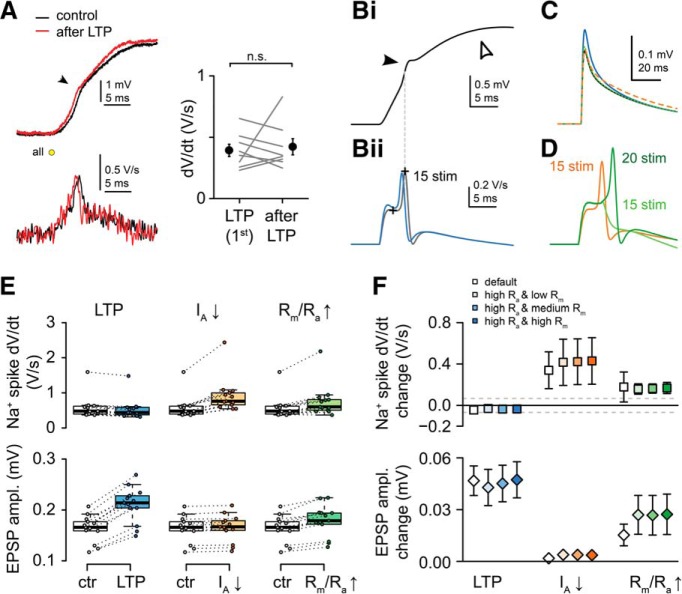
Changes in dendritic excitability by the LTP induction protocol cannot explain somatic increase in EPSP amplitude. ***A*** Left, Representative voltage trace (top) and dV/dt (bottom) of the dendritic Na^+^ spike evoked by the first stimulus of the LTP induction protocol (black), and >40 min after the LTP induction (red). Arrow points to fast spikelets. Right, Summary of 8 similar recordings (*p* = 0.67, Wilcoxon test). Filled symbols and error bars represent mean ± SEM. ***B–E***, Simulations in a biophysical CA1PC model. ***Bi***, Somatic membrane potential response to stimulation of 15 synapses (ISD: 1 μm, dt = 0.3 ms) on a terminal basal dendritic branch. Note the fast Na^+^ (filled arrowhead) and the slower NMDA-spikes (open arrowhead). ***Bii***, dV/dt of the somatic voltage response in control condition (black) and after increasing AMPA conductance of the synapses from 0.6 to 0.84 nS (blue; NMDA conductance constant 0.8 nS). The strength of the somatically recorded dendritic Na^+^ spike (measured between the crosses) is not substantially affected. ***C***, Unitary synaptic EPSPs in control (black), after increasing the AMPA conductance (blue; LTP), after decreasing K^+^ channel density to 0 in the stimulated branch (dotted orange; I_A_-down), or after halving R_a_ and quadrupling R_m_ in the stimulated branch (green; R_m_/R_a_-up). ***D***, dV/dt of the somatic voltage responses after changing local excitability through active (I_A_-down, orange) or passive (R_m_/R_a_-up, green) mechanisms. With increased R_m_/R_a_, 15 stimuli failed to elicit dendritic Na^+^ spike (light green), so strength was measured at 20 stimulus (dark green). ***E***, Summary data from 13 simulated basal branches. Only changing the synaptic conductance (LTP) but not changing local dendritic excitability via active (I_A_-down) or passive (R_m_/R_a_-up) mechanisms was consistent with the experimental data (increased EPSP associated with no changes in dV/dt amplitude). In some branches, no Na^+^ spike was triggered after increasing the R_m_/R_a_. Box plots represent median (line), interquartile ranges (box), and the last data point within the 1.5x interquartile range (whiskers). ***F***, Impact of LTP, I_A_-down, and R_m_/R_a_-up conditions using different combinations of varied R_m_/R_a_ parameters (see Materials and Methods). The predicted Na^+^ spike dV/dt changes by I_A_-down and R_m_/R_a_-up conditions are above the threshold of detectability (gray dashed lines indicate SD of the measured dV/dt during the baseline). Symbols and error bars represent mean ± SD. In some cases, error bars are smaller than symbols.

We considered the possibility that the LTP induction protocol, triggering repeated dendritic spikes, perhaps produced a general change in the electrical properties of the stimulated dendrite (Losonczy et al., 2008), leading to a virtual increase of synaptic voltage signals at the soma. However, the somatic strength of dendritic Na^+^ spikes (dV/dt), a parameter expected to increase by enhanced dendritic excitability (Losonczy et al., 2008; Makara et al., 2009), did not systematic change from the value measured during the first pulse of the LTP induction protocol to that evoked again at the end of the experiments (*n* = 8, *p* = 0.67, Wilcoxon test; Fig. 7*A*). To further explore whether changes in the dendritic excitability can explain our data, we implemented a detailed biophysical model of a CA1PC (Ujfalussy and Makara, 2020) and measured the somatic response amplitude to near-synchronous stimulation of 1–30 excitatory synaptic inputs (see Materials and Methods). In agreement with the experimental data, sufficiently strong stimulations elicited local dendritic Na^+^ and NMDA spikes in the biophysical model, visible as small fast spikelets and slow plateaus, respectively, in the soma (Fig. 7*B*). We used this model to explore which mechanisms can increase synaptic EPSP amplitudes without changing the strength of the Na^+^ spikelets as measured in the soma. Increasing the local excitability of the branch by changing passive parameters (increasing the local membrane resistivity [R_m_] and decreasing axial resistivity [R_a_]) within the branch increased the amplitude of individual EPSPs, but it also significantly increased dV/dt of the somatic spikelets (Fig. 7*C–E*). Changing the local excitability by locally eliminating K^+^ channels also increased dV/dt of the somatic spikelets but failed to increase EPSP amplitudes (Fig. 7*C–E*). On the other hand, increasing the AMPA conductance of the synapses by 40% (mimicking LTP) increased the amplitude of the EPSPs without changing the spikelets (Fig. 7*B–E*). These effects were robust against changing the passive parameters in the model (Fig. 7*F*). These simulations made it unlikely that changes in dendritic excitability by so far described mechanisms could alone explain the increase in somatically measured amplitude of the test spine EPSPs, and suggest that crosstalk was most likely mediated by synaptic mechanisms.

**Figure 8.**
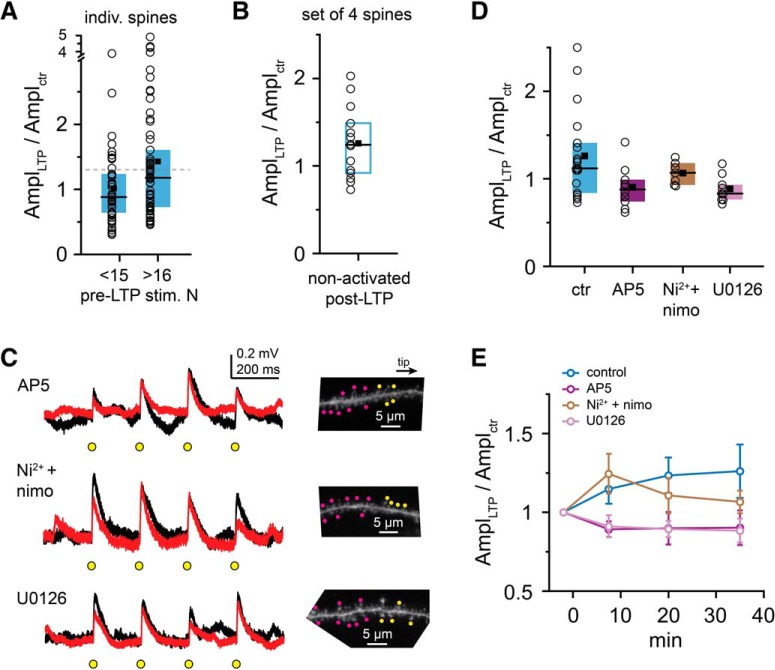
Mechanisms involved in heterosynaptic crosstalk. ***A***, Comparison of EPSP amplitude change in individual test spines that were stimulated ≤15× and ≥16× before the heterosynaptic LTP protocol. Dashed gray line indicates 30% increase. ***B***, EPSP amplitude change measured 30–40 min after the heterosynaptic LTP protocol, with postinduction test stimulation suspended for the first 30 min. ***C***, Representative experiments (black represents baseline; red represents >30 min after LTP induction) with bath application of APV (50 μm, top), Ni^2+^ plus nimodipine (10 and 100 μm, respectively, middle), or U0126 (20 μm, bottom). ***D***, Effect of inhibiting NMDA receptors (AP5), T-, R-, and L-type VGCCs (Ni^2+^ + nimodipine), or MEK/ERK pathway (U0126) on crosstalk plasticity. Test spines were located distally from LTP induction spines in all experiments testing different pharmacological conditions (control group includes the experiments with positive distances in Fig. 6*C*). ***E***, Time course of mean EPSP changes in heterosynaptic experiments under different pharmacological conditions. There is a transient increase of EPSP amplitudes in VGCC inhibitors.

Previous studies showed that LTP at a single spine can lower LTP induction threshold at nearby spines for several minutes, so that even weak stimuli can induce potentiation (Harvey and Svoboda, 2007; Hedrick et al., 2016). Thus, we asked whether the crosstalk plasticity may be related to the weak test stimuli applied to monitor EPSPs. Since the initial selection of the four test spines involved variable numbers of pre-LTP stimuli at different spines (see Materials and Methods), we first analyzed whether pre-LTP stimulation was related to the ability of spines to develop potentiation. Although we did not find a correlation between the number of pre-LTP stimuli and the magnitude of LTP by the individual spines (Spearman *r* = 0.096, *p* = 0.308, *n* = 113 spines), when we separated test spines based on the number of received pre-LTP stimuli into two groups divided near the median (16 stimuli, range: 6–43), we found a trend for smaller EPSP amplitude change and fewer potentiated spines in the spine group receiving ≤15 pre-LTP stimuli (1.02 ± 0.08, *n* = 50; 22% of test spines potentiated) than in those receiving ≥16 pre-LTP stimuli (1.43 ± 0.13, *n* = 63; *p* = 0.022, Mann–Whitney test, 40% of test spines potentiated, *p* = 0.045, χ^2^ test; Fig. 8*A*). This raises the possibility that synapse activation before LTP induction by other synapses may facilitate crosstalk potentiation. Suspending test stimulation for 30 min after LTP induction did not eliminate the crosstalk (EPSP amplitude measured at 30–40 min: 1.26 ± 0.11, *n* = 14 experiments, 42% of test spines potentiated; Fig. 8*B*).

### Biophysical mechanism of crosstalk

The crosstalk mechanism was NMDAR-dependent because no potentiation developed in the presence of d-AP5 (50 μm; Fig. 8*C–E*; EPSP amplitude: 0.90 ± 0.07, *n* = 10 experiments, *p* = 0.023, significant by Holm-Bonferroni-corrected α with Mann–Whitney tests after Kruskal–Wallis test with *p* = 0.022 for control, AP5, U0126, and VGCC blocker groups; see below). Because independent Ca^2+^ measurements showed substantial Ca^2+^ influx in test spines by d-spikes evoked during the heterosynaptic LTP induction protocol (data not shown) most likely via VGCCs (Losonczy and Magee, 2006; Losonczy et al., 2008), and because VGCCs can play a role in some forms of LTP at CA3-CA1 synapses (Tigaret et al., 2016), we tested whether VGCCs are important for crosstalk plasticity. In the presence of T-, R-, and L-type VGCC inhibitors (100 μm Ni^2+^ and 10 μm nimodipine) (Harnett et al., 2012; Weber et al., 2016), we observed an initial increase in EPSP amplitude in test spines, followed by gradual decline toward the baseline (Fig. 8*C–E*; average EPSP amplitude at 7.5 min after LTP protocol: 1.26 ± 0.07; at 30–40 min: 1.07 ± 0.05, *n* = 8, *p* = 0.042, Wilcoxon test between time points), but the EPSP amplitude change at 30–40 min was not significantly different from that in ACSF (*p* = 0.422, Mann–Whitney test). Thus, VGCCs are not indispensable for crosstalk, but they may support stabilization of the process. Finally, inhibition of the MEK/ERK pathway by U0126 (Harvey et al., 2008; Tang and Yasuda, 2017), which was proposed to mediate local metaplasticity of nearby spines via small GTPases (Harvey and Svoboda, 2007; Harvey et al., 2008) eliminated the crosstalk (test spine EPSP amplitude at 30–40 min in 20 μm U0126: 0.88 ± 0.05, *n* = 9, *p* = 0.011, significant by Holm-Bonferroni-corrected α with Mann–Whitney test; Fig. 8*C–E*). Together, the results suggest that crosstalk potentiation of synapses by nearby activity patterns evoking d-spikes may provide a less effective, but not negligible, mechanism to increase synaptic strength, via signaling mediated by NMDARs and MEK/ERK activation.

## Discussion

Using combined 2PI, 2PGU, and electrophysiology, we explored how the fine-grained spatial pattern and the form of voltage integration determines plasticity of different excitatory synaptic input patterns along individual dendrites. We demonstrate that several mechanisms can facilitate local, spatially structured forms of LTP by correlated inputs depending on the fine structure of the input pattern. Since the spatial input requirements of these mechanisms vary, their combination may well explain the rich forms of spatially structured LTP observed in *in vivo* experiments. First, subthreshold cooperative LTP can copotentiate coactive synapses that are in close proximity to each other; this form of interaction is efficient only in relatively distal dendritic segments, such as terminal dendrites. Second, input patterns that evoke d-spikes are strengthened regardless of their fine-scale spatial arrangement and location; in this case, it is likely that the propagation capacity of the d-spike (Losonczy et al., 2008) will determine the size of the plasticity compartment that can potentially extend to an entire branch. Third, we find that strong input patterns activating d-spikes can induce crosstalk plasticity in nearby, nonsynchronous synapses.

In this and a previous report (Weber et al., 2016), we have demonstrated that distal segments of thin dendrites (where synaptically evoked dendritic voltage signals are large due to high impedance; Rinzel and Rall, 1974; Harnett et al., 2012) provide favorable environment for even low numbers (≥3) of clustered inputs to cooperate and copotentiate without generating d-spikes. This mechanism is input specific and is efficient only when the coactive synapses are located within ∼5–10 μm distance; it is possible that biochemical compartmentalization contributes to constraining the spatial limits for subthreshold LTP. This small number and spatial scale is remarkably consistent with that of small functional spine clusters observed in several PC types, including CA1 (Sheffield et al., 2017) and cortical PCs (Kleindienst et al., 2011; Takahashi et al., 2012; Iacaruso et al., 2017; Scholl et al., 2017; Kerlin et al., 2019; Lee et al., 2019). Such small clusters are neither necessary (Losonczy and Magee, 2006) nor sufficient to evoke d-spikes even under simulated *in vivo* conditions (Ujfalussy and Makara, 2020), but this is actually not needed for their local plasticity by the mechanism we describe. Notably, in the absence of a substantial voltage nonlinearity in EPSP integration, activation of such small distal synapse clusters will not bias somatic output until potentiation developed (Ujfalussy and Makara, 2020); therefore, the occurrence of clustered activity might be overlooked or underestimated by somatic recording.

In addition to subthreshold LTP, our results also support a role for local d-spikes (evoked by larger input groups) to induce LTP, in accordance with previous work (Golding et al., 2002; Remy and Spruston, 2007; Kim et al., 2015). In contrast to subthreshold LTP, this mechanism does not critically depend on the precise dendritic location and fine spatial arrangement of the synapses involved (albeit the input threshold to evoke d-spikes is lower distally, see Losonczy and Magee, 2006; Katz et al., 2009; Branco and Häusser, 2011), and can be triggered by just a few sufficiently strong activity events. LTP induced by regenerative d-spikes may thus support rapid and branch-specific (rather than tightly clustered) synaptic plasticity (Yang et al., 2014; Cichon and Gan, 2015; Zhang et al., 2015) and connectivity (Druckmann et al., 2014; Lee et al., 2019).

The above results highlight fundamental location-dependent differences in synaptic learning rules, even in a single dendritic branch. For LTP at proximal dendritic segments, regenerative dendritic events seem essential. Because the local threshold of d-spike generation is relatively high in proximal low-impedance dendritic segments, proximal synapses potentiate most likely if they are coactive with strong input patterns distributed throughout the dendrite (to elicit d-spikes) or throughout the cell (to elicit APs). In contrast, at distal dendritic locations, cooperative LTP can occur by relatively small numbers of inputs both by subthreshold and d-spike-mediated mechanisms, in line with observation of higher cluster density at distal dendritic segments evoked *in vivo* by sensory experience (Makino and Malinow, 2011). If LTP is followed by consolidation and persistence of the synapse (Hill and Zito, 2013), these location-dependent plasticity rules may affect synaptic connectivity (Bono and Clopath, 2017) and may result in distance-dependent bias in synaptic tuning properties (Iacaruso et al., 2017; Scholl et al., 2017), for example, proximal inputs more likely cotuned with the soma and distal inputs more likely cotuned with their neighbors (Iacaruso et al., 2017; but see Scholl et al., 2017). Subthreshold and suprathreshold LTP may also be hierarchically organized so that initial gradual potentiation of repeatedly activated small distal input clusters would help to reach d-spike threshold, recruiting a second, spatially less constrained and faster mechanism that may eventually also evoke somatic AP firing activating global Hebbian synaptic plasticity.

In addition to cooperative LTP of synchronized inputs, we observed less prominent but not negligible heterosynaptic potentiation of inputs in the vicinity of synapses evoking d-spikes. While Hebbian LTP was postulated to be input-specific at large scale, several heterosynaptic plasticity forms have been demonstrated locally in dendrites, including not only depression (Oh et al., 2015; El-Boustani et al., 2018) but also potentiation (Harvey and Svoboda, 2007; Govindarajan et al., 2011; Murakoshi et al., 2011; Hedrick et al., 2016). Specifically, LTP of some synapses may promote potentiation of other, weakly and nonsynchronously active nearby synapses via diffusion of small GTPases, on up to few tens of micrometers and minutes spatiotemporal distance (Harvey and Svoboda, 2007; Govindarajan et al., 2011; Murakoshi et al., 2011; Hedrick et al., 2016). It is tempting to speculate that the crosstalk plasticity we observed may have a similar mechanism: e.g., synaptic activation by test stimuli (in our case, ∼1.5 min earlier) may prime test spines for local plasticity crosstalk from LTP-induction spines, mediated via the MEK/ERK pathway. Since measuring plasticity of unstimulated synapses is not feasible with our experimental method, it is difficult to determine to what extent crosstalk plasticity depends on activity; although our analysis suggests a relationship, further experiments with alternative techniques will be needed to address this question more extensively.

What can be the role of plasticity crosstalk in information coding? Modification of synapse strength by activity of other inputs may seem unfavorable at first sight due to degradation of input specificity. However, integrated storage of synaptic information representing events that occur within a time window of minutes may be behaviorally relevant, as it could bind temporally separate yet associated components (including less salient ones) of a complex experience onto a dendritic segment (Govindarajan et al., 2006; Wiegert and Oertner, 2015; Kastellakis et al., 2016), allowing subsequent simultaneous recurrence of the components to be retrieved more efficiently through dendritic amplification. In addition, since plasticity crosstalk is local, it would only affect segments receiving robust local input. Supporting the relevance of mechanisms promoting copotentiation of temporally separated inputs, another unorthodox form of LTP has been recently described in CA1PCs that is induced by long dendritic plateau potentials and strengthens inputs that were active a short interval (few seconds) earlier or later (Bittner et al., 2017).

In conclusion, our results reveal a large room for cooperative synaptic plasticity to occur locally in dendrites without somatic output, allowing even silent neurons to store information. The fine-scale distribution of active synapses and the local electrical properties of the dendrites, together with other conventional plasticity rules (Clopath et al., 2010; Feldman, 2012), can determine the capacity of input patterns to evoke long-term plasticity. The preferential strengthening (and possibly persistence) of specific arrangements of synaptic connections may contribute to experience-related emergence and refinement of neuronal tuning (Sheffield et al., 2017), and ultimately to the creation of highly specific synaptic engrams of memory traces (Govindarajan et al., 2006). Deciphering the local biophysical processes of synaptic plasticity is not only necessary to understand the computational principles underlying the development of behaviorally relevant and flexible representations by cortical circuits, but may also help to achieve more powerful artificial learning algorithms paralleling the performance of the living brain.

## References

[B1] Bittner KC, Milstein AD, Grienberger C, Romani S, Magee JC (2017) Behavioral time scale synaptic plasticity underlies CA1 place fields. Science 357:1033–1036. 10.1126/science.aan3846 28883072 PMC7289271

[B2] Bono J, Clopath C (2017) Modeling somatic and dendritic spike mediated plasticity at the single neuron and network level. Nat Commun 8:706. 10.1038/s41467-017-00740-z 28951585 PMC5615054

[B3] Branco T, Häusser M (2011) Synaptic integration gradients in single cortical pyramidal cell dendrites. Neuron 69:885–892. 10.1016/j.neuron.2011.02.006 21382549 PMC6420135

[B4] Cichon J, Gan WB (2015) Branch-specific dendritic Ca(2+) spikes cause persistent synaptic plasticity. Nature 520:180–185. 10.1038/nature14251 25822789 PMC4476301

[B5] Clopath C, Büsing L, Vasilaki E, Gerstner W (2010) Connectivity reflects coding: a model of voltage-based STDP with homeostasis. Nat Neurosci 13:344–352. 10.1038/nn.2479 20098420

[B6] Druckmann S, Feng L, Lee B, Yook C, Zhao T, Magee JC, Kim J (2014) Structured synaptic connectivity between hippocampal regions. Neuron 81:629–640. 10.1016/j.neuron.2013.11.026 24412418

[B7] El-Boustani S, Ip JP, Breton-Provencher V, Knott GW, Okuno H, Bito H, Sur M (2018) Locally coordinated synaptic plasticity of visual cortex neurons in vivo. Science 360:1349–1354. 10.1126/science.aao0862 29930137 PMC6366621

[B8] Feldman DE (2012) The spike-timing dependence of plasticity. Neuron 75:556–571. 10.1016/j.neuron.2012.08.001 22920249 PMC3431193

[B9] Frank AC, Huang S, Zhou M, Gdalyahu A, Kastellakis G, Silva TK, Lu E, Wen X, Poirazi P, Trachtenberg JT, Silva AJ (2018) Hotspots of dendritic spine turnover facilitate clustered spine addition and learning and memory. Nat Commun 9:422. 10.1038/s41467-017-02751-2 29379017 PMC5789055

[B10] Fu M, Yu X, Lu J, Zuo Y (2012) Repetitive motor learning induces coordinated formation of clustered dendritic spines in vivo. Nature 483:92–95. 10.1038/nature10844 22343892 PMC3292711

[B11] Golding NL, Staff NP, Spruston N (2002) Dendritic spikes as a mechanism for cooperative long-term potentiation. Nature 418:326–331. 10.1038/nature00854 12124625

[B12] Gordon U, Polsky A, Schiller J (2006) Plasticity compartments in basal dendrites of neocortical pyramidal neurons. J Neurosci 26:12717–12726. 10.1523/JNEUROSCI.3502-06.2006 17151275 PMC6674852

[B13] Govindarajan A, Kelleher RJ, Tonegawa S (2006) A clustered plasticity model of long-term memory engrams. Nat Rev Neurosci 7:575–583. 10.1038/nrn1937 16791146

[B14] Govindarajan A, Israely I, Huang SY, Tonegawa S (2011) The dendritic branch is the preferred integrative unit for protein synthesis-dependent LTP. Neuron 69:132–146. 10.1016/j.neuron.2010.12.008 21220104 PMC3032443

[B15] Harnett MT, Makara JK, Spruston N, Kath WL, Magee JC (2012) Synaptic amplification by dendritic spines enhances input cooperativity. Nature 491:599–602. 10.1038/nature11554 23103868 PMC3504647

[B16] Harvey CD, Svoboda K (2007) Locally dynamic synaptic learning rules in pyramidal neuron dendrites. Nature 450:1195–1200. 10.1038/nature06416 18097401 PMC3425382

[B17] Harvey CD, Yasuda R, Zhong H, Svoboda K (2008) The spread of ras activity triggered by activation of a single dendritic spine. Science 321:136–140. 10.1126/science.1159675 18556515 PMC2745709

[B18] Hedrick NG, Harward SC, Hall CE, Murakoshi H, McNamara JO, Yasuda R (2016) Rho GTPase complementation underlies BDNF-dependent homo- and heterosynaptic plasticity. Nature 538:104–108. 10.1038/nature19784 27680697 PMC5361895

[B19] Hill TC, Zito K (2013) LTP-induced long-term stabilization of individual nascent dendritic spines. J Neurosci 33:678–686. 10.1523/JNEUROSCI.1404-12.2013 23303946 PMC6704923

[B20] Iacaruso MF, Gasler IT, Hofer SB (2017) Synaptic organization of visual space in primary visual cortex. Nature 547:449–452. 10.1038/nature23019 28700575 PMC5533220

[B21] Jarsky T, Roxin A, Kath WL, Spruston N (2005) Conditional dendritic spike propagation following distal synaptic activation of hippocampal CA1 pyramidal neurons. Nat Neurosci 8:1667–1676. 10.1038/nn1599 16299501

[B22] Kastellakis G, Silva AJ, Poirazi P (2016) Linking memories across time via neuronal and dendritic overlaps in model neurons with active dendrites. Cell Rep 17:1491–1504. 10.1016/j.celrep.2016.10.015 27806290 PMC5149530

[B23] Katz Y, Menon V, Nicholson DA, Geinisman Y, Kath WL, Spruston N (2009) Synapse distribution suggests a two-stage model of dendritic integration in CA1 pyramidal neurons. Neuron 63:171–177. 10.1016/j.neuron.2009.06.023 19640476 PMC2921807

[B24] Kerlin A, Boaz M, Flickinger D, MacLennan BJ, Dean MB, Davis C, Spruston N, Svoboda K (2019) Functional clustering of dendritic activity during decision-making. eLife 8:e46966. 10.7554/eLife.46966 31663507 PMC6821494

[B25] Kim Y, Hsu CL, Cembrowski MS, Mensh BD, Spruston N (2015) Dendritic sodium spikes are required for long-term potentiation at distal synapses on hippocampal pyramidal neurons. eLife 4:e06414. 10.7554/eLife.06414 26247712 PMC4576155

[B26] Kleindienst T, Winnubst J, Roth-Alpermann C, Bonhoeffer T, Lohmann C (2011) Activity-dependent clustering of functional synaptic inputs on developing hippocampal dendrites. Neuron 72:1012–1024. 10.1016/j.neuron.2011.10.015 22196336

[B27] Larkum ME, Nevian T (2008) Synaptic clustering by dendritic signalling mechanisms. Curr Opin Neurobiol 18:321–331. 10.1016/j.conb.2008.08.013 18804167

[B28] Larkum ME, Nevian T, Sandler M, Polsky A, Schiller J (2009) Synaptic integration in tuft dendrites of layer 5 pyramidal neurons: a new unifying principle. Science 325:756–760. 10.1126/science.1171958 19661433

[B29] Lee KS, Vandemark K, Mezey D, Shultz N, Fitzpatrick D (2019) Functional synaptic architecture of callosal inputs in mouse primary visual cortex. Neuron 101:421–428. 10.1016/j.neuron.2018.12.005 30658859 PMC7012385

[B30] Losonczy A, Magee JC (2006) Integrative properties of radial oblique dendrites in hippocampal CA1 pyramidal neurons. Neuron 50:291–307. 10.1016/j.neuron.2006.03.016 16630839

[B31] Losonczy A, Makara JK, Magee JC (2008) Compartmentalized dendritic plasticity and input feature storage in neurons. Nature 452:436–441. 10.1038/nature06725 18368112

[B32] Makara JK, Losonczy A, Wen Q, Magee JC (2009) Experience-dependent compartmentalized dendritic plasticity in rat hippocampal CA1 pyramidal neurons. Nat Neurosci 12:1485–1487. 10.1038/nn.2428 19898470

[B33] Makino H, Malinow R (2011) Compartmentalized versus global synaptic plasticity on dendrites controlled by experience. Neuron 72:1001–1011. 10.1016/j.neuron.2011.09.036 22196335 PMC3310180

[B34] Matsuzaki M, Honkura N, Ellis-Davies GC, Kasai H (2004) Structural basis of long-term potentiation in single dendritic spines. Nature 429:761–766. 10.1038/nature02617 15190253 PMC4158816

[B35] Murakoshi H, Wang H, Yasuda R (2011) Local, persistent activation of rho GTPases during plasticity of single dendritic spines. Nature 472:100–104. 10.1038/nature09823 21423166 PMC3105377

[B36] Oh WC, Parajuli LK, Zito K (2015) Heterosynaptic structural plasticity on local dendritic segments of hippocampal CA1 neurons. Cell Rep 10:162–169. 10.1016/j.celrep.2014.12.016 25558061 PMC4294981

[B37] Polsky A, Mel BW, Schiller J (2004) Computational subunits in thin dendrites of pyramidal cells. Nat Neurosci 7:621–627. 10.1038/nn1253 15156147

[B38] Redondo RL, Morris RG (2011) Making memories last: the synaptic tagging and capture hypothesis. Nat Rev Neurosci 12:17–30. 10.1038/nrn2963 21170072

[B39] Remy S, Spruston N (2007) Dendritic spikes induce single-burst long-term potentiation. Proc Natl Acad Sci U S A 104:17192–17197. 10.1073/pnas.0707919104 17940015 PMC2040482

[B40] Remy S, Csicsvari J, Beck H (2009) Activity-dependent control of neuronal output by local and global dendritic spike attenuation. Neuron 61:906–916. 10.1016/j.neuron.2009.01.032 19323999

[B41] Rinzel J, Rall W (1974) Transient response in a dendritic neuron model for current injected at one branch. Biophys. J 14:759–790. 10.1016/S0006-3495(74)85948-5 4424185 PMC1334571

[B42] Scholl B, Wilson DE, Fitzpatrick D (2017) Local order within global disorder: synaptic architecture of visual space. Neuron 96:1127–1138. 10.1016/j.neuron.2017.10.017 29103806 PMC5868972

[B43] Sheffield ME, Adoff MD, Dombeck DA (2017) Increased prevalence of calcium transients across the dendritic arbor during place field formation. Neuron 96:490–504. 10.1016/j.neuron.2017.09.029 29024668 PMC5642299

[B44] Takahashi N, Kitamura K, Matsuo N, Mayford M, Kano M, Matsuki N, Ikegaya Y (2012) Locally synchronized synaptic inputs. Science 335:353–356. 10.1126/science.1210362 22267814

[B45] Tang S, Yasuda R (2017) Imaging ERK and PKA activation in single dendritic spines during structural plasticity. Neuron 93:1315–1324. 10.1016/j.neuron.2017.02.032 28285819 PMC6042854

[B46] Tigaret CM, Olivo V, Sadowski JH, Ashby MC, Mellor JR (2016) Coordinated activation of distinct Ca(2+) sources and metabotropic glutamate receptors encodes Hebbian synaptic plasticity. Nat Commun 7:10289 10.1038/ncomms10289 26758963 PMC4735496

[B47] Ujfalussy BB, Makara JK (2020) Impact of functional synapse clusters on neuronal response selectivity. Nat Commun 11:1413. 32179739 10.1038/s41467-020-15147-6 PMC7075899

[B48] Weber JP, Andrásfalvy BK, Polito M, Magó Á, Ujfalussy BB, Makara JK (2016) Location-dependent synaptic plasticity rules by dendritic spine cooperativity. Nat Commun 7:11380. 10.1038/ncomms11380 27098773 PMC4844677

[B49] Wiegert JS, Oertner TG (2015) Neighborly synapses help each other out. Nat Neurosci 18:326–327. 10.1038/nn.3955 25710831

[B50] Winnubst J, Cheyne JE, Niculescu D, Lohmann C (2015) Spontaneous activity drives local synaptic plasticity in vivo. Neuron 87:399–410. 10.1016/j.neuron.2015.06.029 26182421

[B51] Yang G, Lai CS, Cichon J, Ma L, Li W, Gan WB (2014) Sleep promotes branch-specific formation of dendritic spines after learning. Science 344:1173–1178. 10.1126/science.1249098 24904169 PMC4447313

[B52] Zhang Y, Cudmore RH, Lin DT, Linden DJ, Huganir RL (2015) Visualization of NMDA receptor-dependent AMPA receptor synaptic plasticity in vivo. Nat Neurosci 18:402–407. 10.1038/nn.3936 25643295 PMC4339371

